# Sliding mode control strategy based on disturbance observer for permanent magnet in-wheel motor

**DOI:** 10.1038/s41598-024-66477-0

**Published:** 2024-07-12

**Authors:** Hao Huang, Chunfeng Yu, Zhonghua Sun, Yuanfeng Zhang, Zhibin Zhao

**Affiliations:** Qingdao Campus, Naval Aviation University, Qingdao, 266000 China

**Keywords:** Electrical and electronic engineering, Mechanical engineering

## Abstract

A novel sliding mode control(NSMC) strategy combined with a fast terminal sliding mode observer(FTSMO) is suggested in this paper to solve the parameter variation issue of permanent magnet in-wheel motor(PMIWM) installed in the distributed drive electrical vehicle (DDEV). First, a novel sliding mode power converging law is employed to enhance the response speed of the PMIWM controller. Second, an FTSMO is suggested to compensate for the parameter variation of the PMIWM system to strengthen the robustness of the control object. Finally, a fuzzy controller is designed to adjust the control parameters of the NSMC to optimize the control performance. Several simulations and experiments demonstrate that the proposed FTSMO-NSMC scheme can precisely compensate for parameter variation of the control object and improve control accuracy effectively.

## Introduction

The electrical vehicle (EV) is gradually becoming the research highlight due to the increasing requirement for energy-saving and environmentally friendly automobiles^1^. Distributed drive electrical vehicle (DDEV) is a novel form of EV that can remove some redundant engine parts such as gearbox, differential, and transmission shaft, which can shorten the transmission chain and enhance the transmission efficiency of vehicles^2^. In addition, DDEV also has the merits of abundant space utilization, low energy consumption rate, and ideal control accuracy, which leads to the brilliant prospects of DDEV in the vehicle industry^3,4^.

To achieve higher transmission efficiency and better space layout structure, permanent magnet in-wheel motors(PMIWM) installed in the wheel of DDEV are widely adopted to provide driving power for DDEV^5^. Because the DDEV uses a cable connection instead of a mechanical connection, and there is no differential between the in-wheel motors, the control precision and response speed of PMIWM are substantially improved, which plays a significant role in the safety and comfort of DDEV^6,7^. Regarding PMIWM control, two key issues should be solved: firstly, how to observe and eliminate disturbance of the PMIWM system^8^. Secondly, how to optimize the control precision and response speed of the PMIWM system to ensure that each PMIWM system can respond to the instruction in a given time^9^. Regarding the first issue, the disturbance of PMIWM includes internal parameter variation and external load torque, moment of inertia and viscous damping coefficient are the internal parameters that are most prone to fluctuation and cause disturbances, so it is crucial to observe moment of inertia, viscous damping coefficient, and external load torque to compensate for disturbance^10,11^. Regarding the second issue, we need to adopt an appropriate control method to improve the dynamic performance of the PMIWM control system.

Based on the above questions, several methods have been adopted to implement the estimation of the parameters and load torque of the PMIWM system. Summarize these methods and divide the methods into four categories: parameter adaptation, model reference adaptive system(MRAS), recursive least squares(RLS), and observer-based schemes^12^. The parameter adaptation method uses adaptive law to estimate the parameters. It can adjust the law in real time, but this estimation method is very burdensome for industrial applications and sensitive to adaptive gain parameters^13^. MRAS has been widely applied because of its good dynamic performance and simple implementation, but this method heavily depends on the accuracy of the PMIWM modeling^14,15^. PMIWM is a nonlinear and robust coupling system that makes it hard to construct an exact model for it, so the MRAS is unsuitable for the parameter estimation of PMIWM. The RLS scheme can be applied to adjust the parameters for simple control objects. However, this method needs a long estimation time, and the estimation precision needs to be improved for the PMIWM applied in the DDEV. Therefore, The RLS method is limited in the application of PMIWM^16^.

The observer-based method includes extended-disturbance observer(EDOB) and sliding mode observer(SMO), and this method has recently become the research highlight for parameter estimation due to simple implementation and precision estimation ability^17^. The EDOB scheme adopted a state and load estimator to estimate and compensate for the mechanical parameters and load torque, which can realize optimized disturbance estimation but owes the drawbacks of poor robustness and high sensitivity to the parameter variations^18,19^. The SMO possesses the merits of strong robustness and is insensitive to parameter variations^20^. Therefore, this method has been adopted to estimate the parameters in many industrial occasions. In^21^, Jin proposed an adaptive control strategy based on SMO for PMSM, which can effectively eliminate the position estimation error of the control object. In^22^, Xu and Chen constructed a adaptive sliding mode observer to accurately estimate the reconfiguration state of each subsystem, and this method can improve the control precision and robustness of the control object. Liu and Zhang addressed a position-estimation method based on the sliding-mode observer and the phase-locked loop to realize sensorless control^17^. In^23^, Huang and Tu proposed a nonsingular terminal sliding mode control scheme combined with an SMO, and this method can effectively estimate the external load torque of the PMIWM and improve the anti-jamming ability of the control object. In^24^ Liu utilized the gradient search for both magnitude and phase of the rotor to enhance the control performance. In^25^ Liu and Li proposed a disturbance rejection control method combining robust speed controller and load observer is proposed for low-speed high-torque PMSM.

In previous disturbance estimation methods, SMO is most widely used by researchers. However, the conventional SMO estimation method owns its defect, that is the sliding mode surface cannot converge to the stable point in a finite time. In order to solve this issue, we propose a fast terminal sliding mode observer (FTSMO), which can solve the converging issue by introducing a nonlinear function. In addition, we design a novel sliding mode control (NSMC) scheme that adapts a novel power converging law that can solve the discontinuous term that existed in the traditional SMC to weaken the chattering value, and the control parameters of NSMC can be adjusted by the fuzzy controller in real time to solve the chattering/converge time dilemma. We denote the proposed method as the FTSMO-NSMC algorithm, and the main contributions are as follows:

1. An NSMC method based on the novel power converging law is designed to reduce the chattering value.
2. An FTSMO is proposed to estimate each motor ’s disturbance of internal parameters and external load torque, which can compensate for the influence of disturbance.
3. A fuzzy controller is designed to adjust the gain parameters of the NSMC scheme in real time to obtain the ideal converging speed and slight chattering value.

The whole paper is presented as follows: “Machine modelling” presents a multi-motor structure of DDEV and constructs the mathematical model of PMIWM. In “Controller design”, An NSMC scheme based on the power converging law is employed to obtain the ideal control performance of each PMIWM. In addition, we adopt an FTSMO to estimate the electric parameters and external load torque and study a fuzzy controller to optimize the speed-tracking performance for the NSMC scheme. Numeral co-simulation and physical experiments for single PMIWM and multi-motor systems are implemented in “Simulation and experiments”. “Conclusion” summarizes the whole paper.

## Machine modelling

This section presents a multi-motor drive structure of the DDEV and analyzes this system’s control parts and control flow. In addition, a simplified mathematical model of PMIWM is constructed and analyzed.

### Multi-motor Structure of DDEV

**Figure 1:**
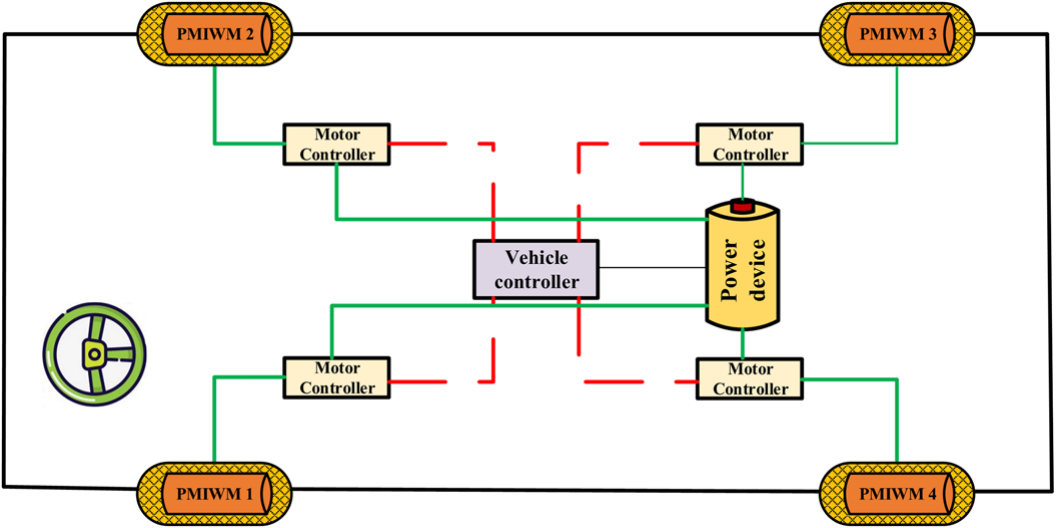
Multi-motor driving structure of DDEV.

The multi-motor driving structure of DDEV is shown in Fig. 1. Compared with the traditional engine vehicle structure, this structure adopts flexible cables to replace the engineering parts to transfer drive power and control signals^26^. In this paper, the front in-wheel motors work as the driving and steering parts for the DDEV, and the rear motors work as the torque-increasing motor device when the vehicle climbs^27^. Therefore, we take the four-in-wheel motor system as the research object to imitate the DDEV’s driving and control in this paper.

In the front in-wheel multi-motor system, because there is no differential between motors, when the DDEV drives on the uneven road surface or turns, the load torque of two front in-wheel motors will be different, which may lead to the unbalanced driving speed of each PMIWM, and further cause the skidding, overturn, and other dangerous accidents of the DDEV.

**Figure 2:**
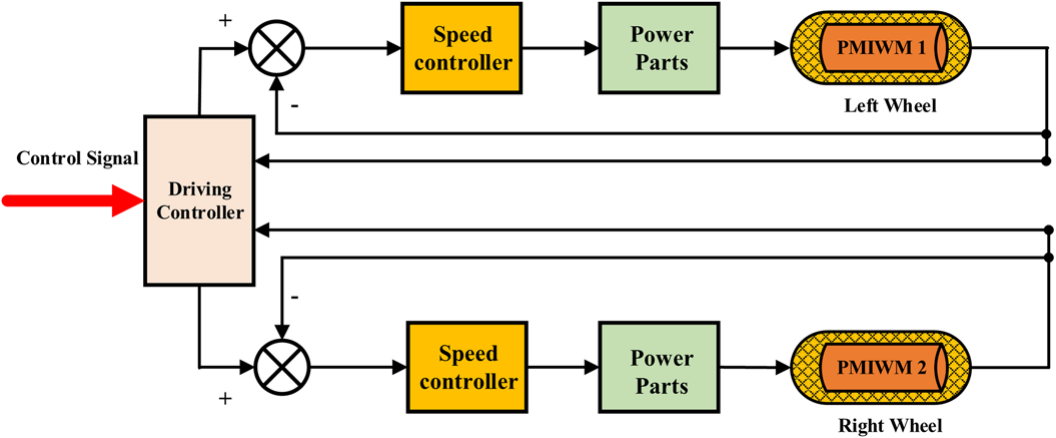
Control diagram of the multi-motor drive system.

The control diagram of the multi-motor driving system is shown in Fig. 2. When the driving controller receives the control signal of the driver, it will send the speed instruction to the speed controller of each PMIWM respectively.

In order to simplify the mathematical model of PMIWM and reduce the computational burden, the following assumptions have been made without influencing the control precision of the motor^28^. (1) The iron loss in the magnetic field is ignored. (2) There is no damping effect on the PMIWM. 3) Surface-mounted installation mode is adopted for the PMIWM, and the inductances of the d-q axis are equal. Based on the above assumptions, the dynamics of a PMIWM in the rotor d-q coordinates can be described as follows:

1 $$\left\{ {\begin{array}{*{20}l} {u_{d} = Ri_{d} - p\omega Li_{q} + L\dot{i}_{d} } \hfill \\ {u_{q} = Ri_{q} + p\omega Li_{d} + p\omega \psi } \hfill \\ \end{array} } \right.$$

2 $$\left\{ {\begin{array}{*{20}l} {T_{e} - T_{L} = J\dot{\omega } + B\omega } \hfill \\ {T_{e} = \frac{3}{2}P\left[ {\left( {L_{d} - L_{q} } \right)i_{d} i_{q} + \psi i_{q} } \right]} \hfill \\ \end{array} } \right.$$

where $i_{d}$ and $i_{q}$ are the currents of *d-q* axis respectively, $L$ is the self-inductance, $L_{d}$ and $L_{q}$ are the inductance of the *d-q* axis respectively, *R* is the stator resistance, $u_{d}$ and $u_{q}$ are the voltage of the *d-q* axis respectively, $\psi$ is permanent magnet flux linkage of motor, $\omega$ is the rotating angular velocity of PMIWM, $T_{e}$ is electromagnetic torque, $T_{L}$ is load torque, $J$ is the moment of inertia, $B$ is viscous friction coefficient.

Based on the assumption 3, $L_{d} { = }L_{q} = L$, the Eq. (2) can be simplified as follows:

3 $$\left\{ {\begin{array}{*{20}l} {T_{e} - T_{L} = J\dot{\omega } + B\omega } \hfill \\ {T_{e} = \frac{3}{2}P\left[ {\left( {L_{d} - L_{q} } \right)i_{d} i_{q} + \psi i_{q} } \right]} \hfill \\ \end{array} } \right.$$

**Figure 3:**
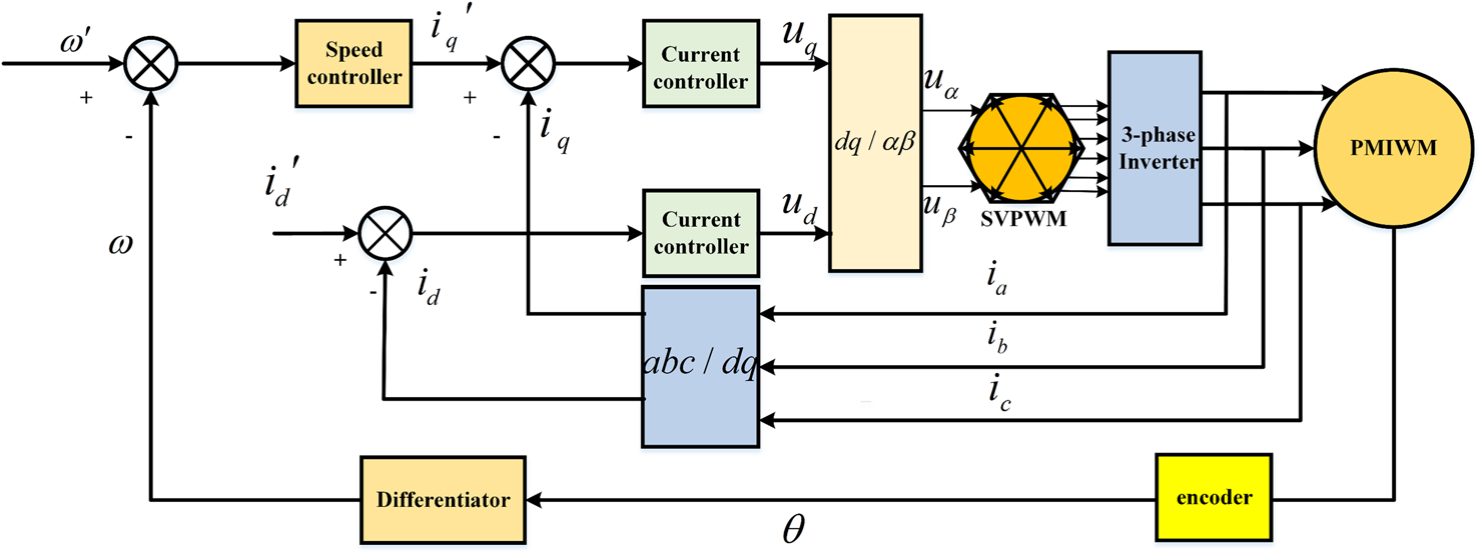
Control layout diagram of the PMIWM system.

Figure 3 presents the control layout diagram of the PMIWM system. Vector control is adopted in this control structure, and the three-phase invert and SVPWM work as the power parts for this control system. The output speed and currents can be calculated through the photoelectric encoder and current sensor, respectively. The motor speed $\omega$, the current $i_{d}$, and the current $i_{q}$ make up closed-loop control parts in the PMIWM control system^29^.

## Controller design

In this section, we propose three schemes to estimate the disturbances and optimize the control performance of the PMIWM system. Firstly, we propose an NSMC scheme to reduce the chattering phenomenon and obtain the ideal converging speed of the sliding mode surface. Secondly, we design an FTSMO to estimate the parameter variation and improve the control accuracy of the PMIWM. Thirdly, we adopt a fuzzy controller and design fuzzy rules to adjust the sliding mode gain of the control strategy in real time.

### NSMC scheme design

The SMC strategy has been widely adopted for the nonlinear control object due to its robustness. However, the chattering phenomenon of the conventional SMC is a significant problem that needs to be solved^30,31^. Some researchers effectively eliminated the chattering phenomenon by reducing the sliding mode gain value, but this method can significantly deteriorate the robustness and control precision of the control system. In order to solve this problem, we adopt a power converging law to replace the exponential converging law of conventional SMC.

In the sliding mode state equation, denote the state parameters as follows:

4 $$\left\{ \begin{gathered} x_{1i} = \omega^{\prime}_{i} - \omega_{i} \hfill \\ x_{2i} = \dot{x}_{1i} = f(x) + gu + b(x) \hfill \\ \end{gathered} \right.i = 1,2$$

where $x_{1i}$ and $x_{2i}$ are state parameters of the *i*th motor in the system, $\omega_{i}^{\prime }$ is the instruction speed of the *i*th motor, and $\omega_{i}$ is the actual speed of the *i*th motor, $u$ is the control output, $g$ is the control parameter of $u$, $b(x)$ is the disturbance.

5 $$s_{i} = cx_{1i}$$

where $s_{i}$ is the sliding mode surface, $c$ is the control parameters of sliding mode surface. In the conventional SMC scheme, the constant rate converging law is expressed as follows:

6 $$\dot{s}_{i} = - \varepsilon {\text{sgn}} (s_{i} ) - ks_{i}$$

where *ε* is the switch gain value, *k* is the sliding mode gain value. Take the derivation of in the Eq. (5) and combine it with Eq. (6), the following equation can be obtained:

7 $$cx_{2i} = - \varepsilon {\text{sgn}} (s_{i} ) - ks_{i}$$

Combine Eq. (7) with Eq. (4), the control output can be expressed as follows:

8 $$u = - g^{ - 1} (x)\left( {f(x) + \varepsilon {\text{sgn}} (s_{i} ) + ks_{i} + b(x)} \right)$$

Equation (8) shows that the discontinuous term ${\text{sgn}} (s_{i} )$ can excite the chattering value of the nonlinear system, which is detrimental to the whole SMC system. In addition, the sliding mode surface’s converging time is determined by the switch gain *ε*. The chattering value will be eliminated if the switch gain decreases, but the reaching time will increase. To solve this chattering value/ reaching time dilemma, we propose an NSMC combined with a novel power converging law, and the novel power converging law of the NSMC is denoted as follows:

9 $$\dot{s}_{i} = - k|s_{i} |^{a} s_{i}$$

where *a* is the power gain of this converging law, *k* > 0,1 > *a* > 0. For the purpose of validating the stability of this NSMC system, construct the Lyapunov function as follows:

10 $$\dot{V} = s_{i} \dot{s}_{i} = s_{i} \cdot ( - k|s_{i} |^{a} )s_{i} = - k|s_{i} |^{a + 2} \le 0$$

From Eq. (10), we can observe that the control system is stable and can converge in a finite time. Combine Eq. (9) with Eq. (4) and the control output *u* can be expressed as follows:

11 $$u = - g^{ - 1} (x)\left( {f(x) - k|s_{i} |^{a} s_{i} + b(x)} \right)$$

Compared Eq. (11) with Eq. (8), the discontinuous term ${\text{sgn}} (s_{i} )$ can be eliminated, which can weaken the chattering value and improve the control precision.

### FTSMO design

Because the PMIWM installed in the DDEV can experience frequent vibration, temperature change, and parts aging, which may cause the parameter variation and load fluctuation of the PMIWM. The main time-variant parameters are the moment of inertia *J*, viscous friction coefficient *B*, and load torque *T*_*L*_^32,33^. Considering the mechanical dynamic model of the PMIWM, a sliding mode observer can be designed as follows:

12 $$\left\{ {\begin{array}{*{20}l} {\dot{\hat{\theta }} = \hat{\omega }} \hfill \\ {\hat{J}\dot{\hat{\omega }} = - \hat{B}\hat{\omega } + T_{e} + u_{0} } \hfill \\ \end{array} } \right.$$

where $\hat{J}$, $\hat{B}$, $\hat{\omega }$, $\dot{\hat{\theta }}$ are the estimation value for the $J$, $B$, $\omega$, $\dot{\hat{\theta }}$, respectively, $u_{0}$ is the observe output of the FTSMO; The mechanical parameters of the PMIWM system can be expressed as follows:

13 $$\left\{ {\begin{array}{*{20}l} {\hat{J} = \Delta \hat{J} + J_{0} } \hfill \\ {\hat{B} = \Delta \hat{B} + B_{0} } \hfill \\ \end{array} } \right.$$

where $J_{0}$ and $B_{0}$ are the initial value of *J* and *B*, respectively. Combine (3) and (12), the error system can be obtained.

as:

14 $$J\dot{\omega } - \hat{J}\dot{\hat{\omega }} = - B\omega - T_{L} + \hat{B}\hat{\omega } - u_{0}$$

Denote $e_{1} = \theta - \hat{\theta }$, and the state equation of FTSMO can be designed as follows:

15 $$\left\{ {\begin{array}{*{20}l} {\dot{e}_{1} = e_{2} } \hfill \\ {\dot{e}_{2} { = }\hat{J}^{ - 1} \left( { - \Delta \hat{J}\dot{\omega }{ + }\left( {\hat{B} - B} \right)\omega - T_{L} - \hat{B}e_{2} - u_{0} } \right)} \hfill \\ \end{array} } \right.$$

Denote the control output *u* as follows:

16 $$\left\{ {\begin{array}{*{20}l} {u_{0} { = }u_{1} { + }u_{2} } \hfill \\ {u_{1} { = }\hat{B}e_{2} } \hfill \\ \end{array} } \right.$$

where $u_{1}$ is the the first compensate value, $u_{2}$ is another compensation value that will be described later. Combined with Eq. (16), Eq. (15) can be expressed as follows:

17 $$\left\{ {\begin{array}{*{20}l} {\dot{e}_{1} = e_{2} } \hfill \\ {\dot{e}_{2} = \hat{J}^{ - 1} \left( { - \Delta \hat{J}\dot{\omega } - \Delta \hat{B}\omega - T_{L} - u_{2} } \right)} \hfill \\ \end{array} } \right.$$

Denote the fast terminal sliding mode surface $s_{2}$ as follows:

18 $$s_{2} = e_{1} + \frac{1}{\beta }e_{2}^{p/q}$$

where *β* is the control parameter of the fast terminal sliding mode surface,* p* and *q* are the power gain of the fast terminal.

sliding mode surface, *p* and *q* are positive odd number and *p* < *q*. Select the Lyapunov function to verify the stability of this FTSMO system:

19 $$\begin{array}{*{20}l} {\dot{V}_{2} = s_{2} \dot{s}_{2} = s_{2} \cdot (e_{2} + \frac{1}{\beta }\frac{p}{q}e_{2}^{p - q/q} \dot{e}_{2} )} \hfill \\ { = s_{2} \cdot \left( {e_{2} + \frac{1}{\beta }\frac{p}{q}e_{2}^{p - q/q} \hat{J}^{ - 1} \left( { - \Delta \hat{J}\dot{\omega } - \Delta \hat{B}\omega - T_{L} + u_{2} } \right)} \right)} \hfill \\ \end{array}$$

***Assumption 1***: The initial mechanical parameters of the PMIWM system satisfy these conditions.

20 $$\left| {\Delta \hat{J}} \right| \le K_{J} ,\left| {\Delta \hat{B}} \right| \le K_{B} ,\left| {T_{L} } \right| \le K_{L}$$

***Assumption 2***: The speed and acceleration of motor are bounded:

21 $$\left| \omega \right| \le K_{a} ,\left| {\dot{\omega }} \right| \le K_{b}$$

Denote the compensation value $u_{2}$ as follows:

22 $$u_{2} = - \left( {K_{J} K_{b} + K_{B} K_{a} + K_{L} + \eta } \right){\text{sgn}}(s_{2} ) + \frac{{B\hat{J}q}}{p}e_{2}^{{\frac{2q - p}{q}}}$$

where $K_{J}$, $K_{b}$, $K_{B}$, $K_{a}$, $K_{L}$ are positive number and the value of them can be obtained through simulation, and positive constant $\eta$ is the compensation boundary. Substituting Eq. (22) into Eq. (19), and the Lyapunov function can be expressed as follows:

23 $$\begin{array}{*{20}l} {\dot{V}_{2} = s_{2} \cdot \left( {e_{2} + \frac{1}{\beta }\frac{p}{q}e_{2}^{p - q/q} \hat{J}^{ - 1} ( - \Delta \hat{J}\dot{\omega } - \Delta \hat{B}\omega - T_{L} + u_{2} )} \right)} \hfill \\ { = s_{2} \cdot \left( {e_{2} + \frac{1}{\beta }\frac{p}{q}e_{2}^{p - q/q} \hat{J}^{ - 1} ( - \Delta \hat{J}\dot{\omega } - \Delta \hat{B}\omega - T_{L} + \left( { - K_{J} K_{a} - K_{B} K_{b} - K_{L} - \eta } \right){\text{sgn}} (s_{2} ) - \frac{{\beta J_{0} q}}{p}e_{2}^{{\frac{2q - p}{q}}} )} \right)} \hfill \\ { = s_{2} \cdot \left( {e_{2} + \frac{1}{\beta }\frac{p}{q}e_{2}^{p - q/q} \hat{J}^{ - 1} \left( {\left( { - K_{J} K_{a} {\text{sgn}} (s_{2} ) - \Delta \hat{J}\dot{\omega }} \right) + \left( { - K_{B} K_{b} {\text{sgn}} (s_{2} ) - \Delta \hat{B}\omega } \right) + \left( { - K_{L} {\text{sgn}} (s_{2} ) - T_{L} } \right) - \eta {\text{sgn}} (s_{2} ) - \frac{{B_{0} J_{0} q}}{p}e_{2}^{{\frac{2q - p}{q}}} } \right)} \right)} \hfill \\ { \le \left( {\left( {s_{2} \cdot \left( { - K_{J} K_{a} {\text{sgn}} (s_{2} ) - \Delta \hat{J}\dot{\omega }} \right) + s_{2} \cdot \left( { - K_{B} K_{b} {\text{sgn}} (s_{2} ) - \Delta \hat{B}\omega } \right) + s_{2} \cdot \left( { - K_{L} {\text{sgn}} (s_{2} ) - T_{L} } \right) - \eta s_{2} \cdot {\text{sgn}} (s_{2} )} \right)\frac{1}{\beta }\frac{p}{q}e_{2}^{p - q/q} \hat{J}^{ - 1} } \right)} \hfill \\ { + \left( {s_{2} \cdot \left( {e_{2} - \left( {\frac{1}{\beta }\frac{p}{q}e_{2}^{p - q/q} \hat{J}^{ - 1} \cdot \frac{{B_{0} \hat{J}_{0} q}}{p}e_{2}^{{\frac{2q - p}{q}}} } \right)} \right)} \right)} \hfill \\ { \le \left( {\left( {s_{2} \cdot \left( { - K_{J} K_{a} {\text{sgn}} (s_{2} ) - \Delta \hat{J}\dot{\omega }} \right) + s_{2} \cdot \left( { - K_{B} K_{b} {\text{sgn}} (s_{2} ) - \Delta \hat{B}\omega } \right) + s_{2} \cdot \left( { - K_{L} {\text{sgn}} (s_{2} ) - T_{L} } \right) - \eta s_{2} \cdot {\text{sgn}} (s_{2} )} \right)\frac{1}{\beta }\frac{p}{q}e_{2}^{p - q/q} \hat{J}^{ - 1} } \right)} \hfill \\ \end{array}$$

From Eq. (20) and Eq. (21), it can be obtained that:

24 $$\left\{ {\begin{array}{*{20}l} {s_{2} \cdot \left( { - K_{J} K_{a} {\text{sgn}} (s_{2} ) - \Delta \hat{J}\dot{\omega }} \right) = - \left| {s_{2} } \right|K_{J} K_{a} - s_{2} \Delta \hat{J}\dot{\omega } \le 0} \hfill \\ {s_{2} \cdot \left( {K_{B} K_{b} {\text{sgn}} (s_{2} ) - \Delta \hat{B}\omega } \right) = - \left| {s_{2} } \right|K_{B} K_{b} - s_{2} \Delta \hat{B}\omega \le 0} \hfill \\ {s_{2} \cdot \left( {K_{L} {\text{sgn}} (s_{2} ) - T_{L} } \right) = - \left| {s_{2} } \right|K_{L} - s_{2} T_{L} \le 0} \hfill \\ {\frac{1}{\beta }\frac{p}{q}e_{2}^{p - q/q} \hat{J}^{ - 1} \ge 0} \hfill \\ \end{array} } \right.$$

From Eq. (24), it can be obtained Eq. (23)meets:

25 $$\dot{V}_{2} \le - \frac{p}{{\beta q\hat{J}}}e_{2}^{p - q/q} \eta \left| {s_{2} } \right| \le 0$$

When the state vector *s*_2_ can converge to $s_{2} = 0$ in finite-time, $e_{2}$ and $\dot{e}_{2}$ in error system will converge to zero along in a finite time. When $e_{2} = 0$ and $\dot{e}_{2} = 0$, the following Equation can be obtained according to Eq. (17):

26 $$- \Delta \hat{J}\dot{\omega }(t) - \Delta \hat{B}\omega (t) - T_{L} (t) - u_{2} (t) = 0$$

From Eq. (25), the estimation of internal parameter disturbance(*B, J*) and external load disturbance($T_{L}$) can be estimated based on ^34^ as follows:

27 $$\left\{ {\begin{array}{*{20}l} {B(t) = B_{0} + \hat{B} = B_{0} + \left( {u_{2} (t - \tau ) - u_{2} (t)} \right)/\left( {\omega (t - \tau ) - \omega (t)} \right)} \hfill \\ {J(t) = J_{0} + \hat{J} = J_{0} + \left( {\left( {u_{2} (t) - u_{2} (t - \tau )} \right)/\dot{\omega }(t) - \dot{\omega }(t - \tau )} \right)} \hfill \\ {T_{L} (t) = - u_{2} (t)} \hfill \\ \end{array} } \right.$$

### Fuzzy controller design

Because the converging speed of the proposed NSMC scheme is based on the power gain a and sliding mode gain k. These two parameters are constant during the control process, which makes it hard to achieve fast converging speed and slight chattering value. In order to solve this problem, we propose a fuzzy controller and design a series of fuzzy rules to adjust the approaching parameters of the NTSM surface online to achieve a balance of eliminating the chattering phenomenon and obtaining an ideal converging speed.

The design process of the fuzzy controller is as follows:

***Step 1*** Denote the sliding mode surface and as the fuzzy input 1 and fuzzy input 2, respectively.

***Step 2*** Denote the approaching parameter and power gain as the fuzzy output 1 and fuzzy output 2, respectively.

***Step 3*** Obtain the approaching parameter and the power gain based on the fuzzy rules based on the NTSM control input and the existing condition of the sliding mode surface. Where $A_{i}^{j}$ is the fuzzy set of input.* i* = 1,2, *j* = 1,2, . . . . . . 5, $B_{i}^{n}$ is the fuzzy set of the conclusion, *i* = 1,2, *n* = 1,2, . . . . . . 25, then the 1–25 fuzzy rules can be obtained as follows:

set of the conclusion, i = 1,2, n = 1,2, . . . . . . 25, then the 1–25 fuzzy rules can be obtained as follows:

- R(1): if *s* is $A_{1}^{1}$ and $\dot{s}$ is $A_{2}^{1}$, then ∆*k* is $B_{1}^{1}$, and ∆*α* is $B_{2}^{1}$.
- R(2): if *s* is $A_{1}^{2}$ and $\dot{s}$ is $A_{2}^{1}$, then ∆*k* is $B_{1}^{1}$, and ∆*α* is $B_{2}^{2}$.
- R(25): if *s* is $A_{1}^{5}$ and $\dot{s}$ is $A_{2}^{5}$, then ∆*k* is $B_{1}^{25}$, and ∆*α* is $B_{2}^{25}$

**Figure 4:**
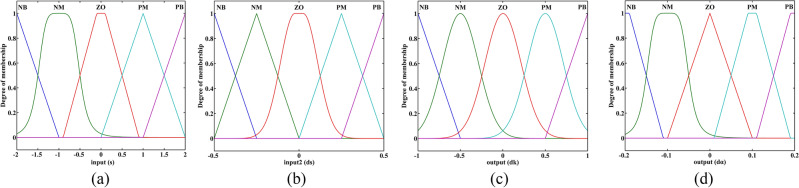
The degree of membership for the fuzzy parameters (**a**) Input *s* (**b**) Input $\dot{s}$ (**c**) Output ∆*k* (**d**) Output ∆*α*.

In this fuzzy control system, *s* and $\dot{s}$ are denoted as inputs for the fuzzy controller; ∆*k* and ∆*α* are denoted as output for the fuzzy controller, the corresponding relation of fuzzy parameters is defined in Table 1 and Table 2, respectively. The degree of membership for the fuzzy parameters is shown in Fig. 4.

**Table 1:** Corresponding relation of fuzzy parameter ∆*k*.

	NB	NM	ZO	PM	PB
NB	NB	NM	ZO	PM	PB
NM	NM	NM	ZO	PM	ZO
ZO	ZO	ZO	ZO	PM	ZO
PM	ZO	PM	PM	PM	PM
PB	PM	PB	PB	PB	PB

**Table 2:** Corresponding relation of fuzzy parameter ∆*α.*

	NB	NM	ZO	PM	PB
NB	NB	NM	NM	NM	NM
NM	NB	NM	NM	NM	ZO
ZO	ZO	ZO	ZO	ZO	ZO
PM	PM	ZO	ZO	PM	PM
PB	PB	PM	PM	PM	PB

## Simulation and experiments

In this section, a simulation system and experimental platform have been established to validate the effectiveness of the proposed FTSMO-NSMC strategy, and the results of the simulation and experiments are presented and discussed in detail.

### Implementation of the simulation

In this subsection, we implement several simulations to verify the control performance of the proposed FTSMO-NSMC strategy. Firstly, we implement the simulations of the parameter crude estimation to demonstrate the estimation accuracy of the proposed FTSMO. Secondly, several speed simulations of the single-motor system have been implemented to present the control performance of the proposed FTSMO-NSMC strategy. Thirdly, we implement the PMIWM system’s loading simulations to test the robustness and anti-interference ability of the proposed scheme. Table 3 and Table 4 presents all the parameters of the PMIWM system for simulation.

**Table 3:** Internal parameters of PMIWM.

Symbol	Definition	Value
*B*	Viscous friction coefficien	0.008N·m·s/rad
*L*_*q*_	Inductance of q axis	8.0mH
*L*_*d*_	Inductance of d axis	8.0mH
*J*	Moment of inertia	0.02 kg·m^2^
*ψ*	Rotor’s magnetic flux	0.295Wb
R	Nominal phase resistance	2.385Ω

**Table 4:** Control parameters of PMIWM.

Symbol	Definition	Value
*α*	Converging law gain	0.008N·m·s/rad
*k*	Sliding mode gain	8.0mH
*β*	Control parameter of FTSMO	8.0mH
*p*	Power gain of FTSMO	0.02 kg·m^2^
*q*	Power gain of FTSMO	0.295Wb
*η*	Compensation boundary	2.385Ω

**Figure 5:**
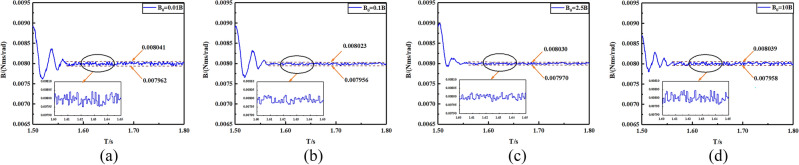
Crude estimation of the viscous friction coefficient *B* (**a**) *B*_0_ = 0.01B (**b**) *B*_0_ = 0.1B (**c**) *B*_0_ = 2.5B (**d**) *B*_0_ = 10B.

**Figure 6:**
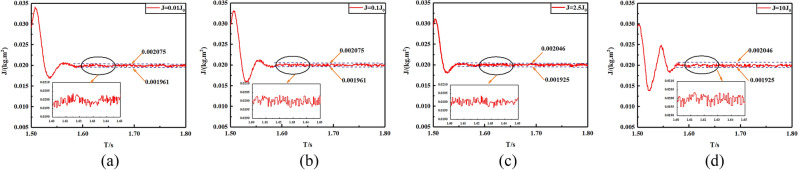
Crude estimation of the moment of inertia *J* (**a**) *J*_0_ = 0.01* J* (**b**) *J*_0_ = 0.1* J* (**c**) *J*_0_ = 2.5* J* (**d**) *J*_0_ = 10* J.*

**Figure 7:**
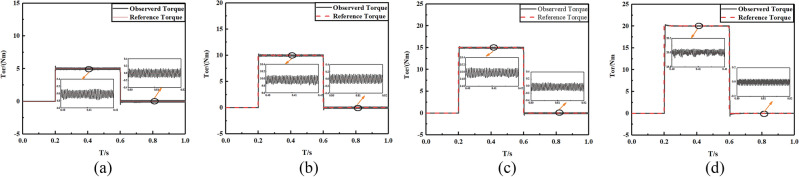
Crude estimation of the load torque *T* (**a**) *T* = 20Nm (**b**) *T* = 15Nm (**c**) *T* = 10Nm (**d**) *T* = 5Nm.

In order to demonstrate the estimation precision of the proposed FTSMO, we implement four different crude estimations of the viscous friction coefficient *B*, which is *B*_0_ = 0.01*B*, 0.1*B*, 2.5*B*, 10*B*, respectively. Figure 5a–d presents the estimation simulation results. It can be seen that these four crude estimation values of the viscous friction coefficient can converge to the actual value within 1.58 s, which presents the estimation precision and rapidity of the proposed FTSMO. In addition, we simulate the estimation value of the moment of inertia *J*, which is *J*_0_ = 0.01* J*,0.1* J*,2.5* J*, 10* J*, and the estimation simulations are shown in Fig. 6a–d. From the simulation results, we can conclude that the proposed FTSMO can observe *J* in finite time with slight fluctuation. We implement the estimation simulations for the load torque T, and the simulation results are shown in Fig. 7a–d. The estimation results show that the proposed FTSMO can track different torque values quickly and precisely. However, Fig. 7 shows some overshooting values during the estimation process, which needs further optimization in future research.

**Figure 8:**
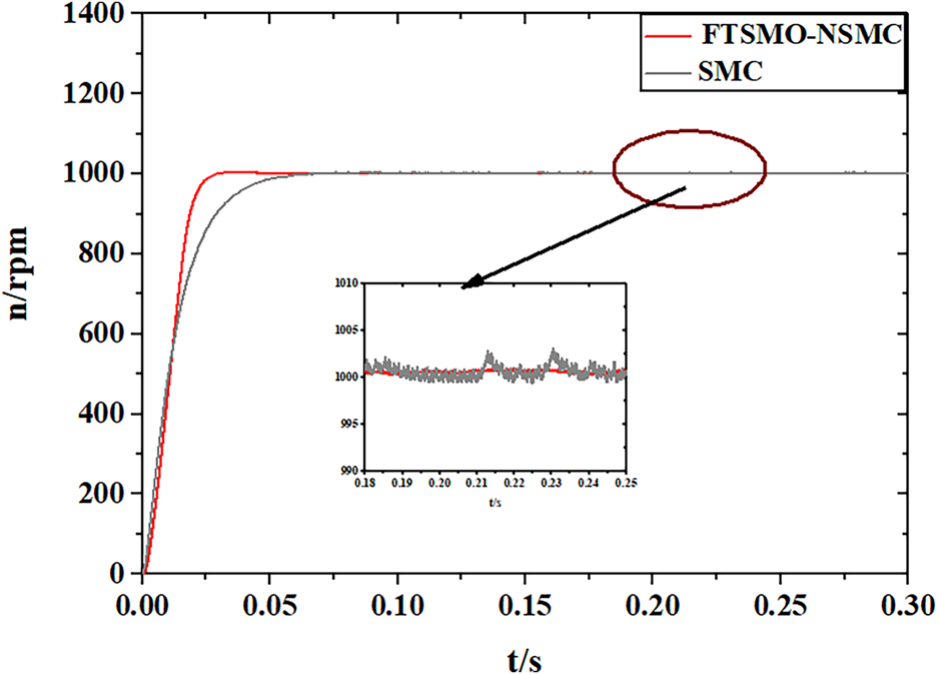
Simulation of the starting Response.

**Figure 9:**
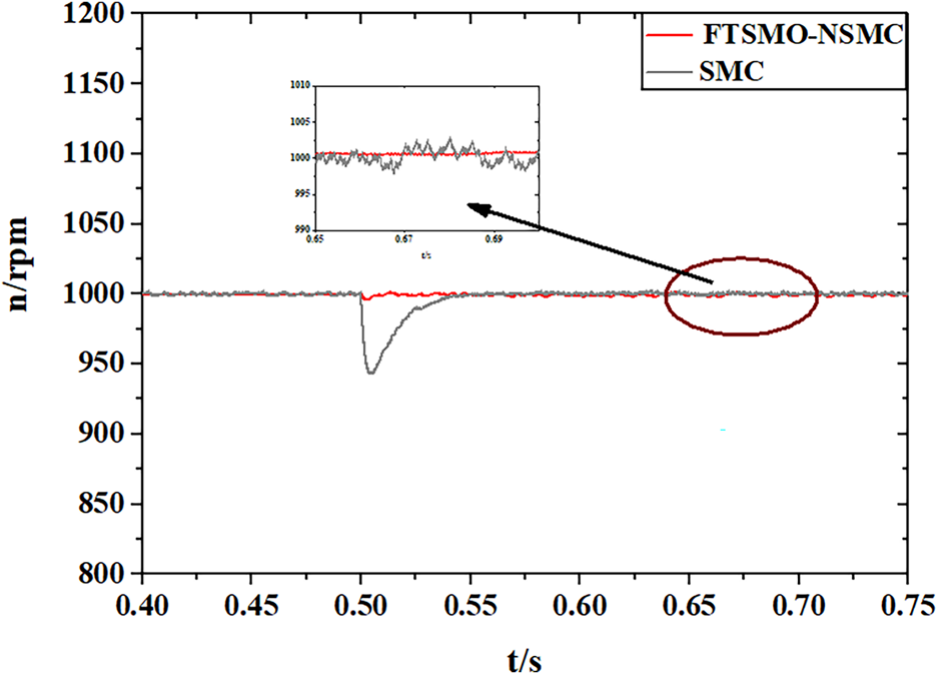
Simulation of the loading response.

After verifying the estimation precision of the proposed FTSMO, we implement starting and loading simulations under the proposed FTSMO-NSMC scheme and conventional SMC scheme, and the simulation results are presented in Figs. 8 and 9, respectively.

As shown in Fig. 8, the proposed FTSMO-NSMC has a quicker starting response and slight fluctuation when reaching the steady state than the conventional SMC scheme. As shown in Fig. 9, the FTSMO-NSMC scheme can respond to load disturbances in a shorter adjustment time. In addition, when the PMIWM system reaches the command speed, the speed fluctuation of the FTSMO-NSMC is around 10 rpm, much smaller than that of the conventional SMC scheme. Table 5 demonstrates the comparison of these two schemes in detail.

**Table 5:** Comparison of the control schemes.

	FTSMO-NSMC	SMC
Startup time (s) (1000 rpm)	0.0367	0.063
Fluctuation value(percent) (1000 rpm)	0.52%	4.23%
Adjust time(10N·m) (s)	0.003	0.038
Adjust fluctuation (10N·m) (percent)	0.43%	3.61%

### Implementation of the experiments

**Figure 10:**
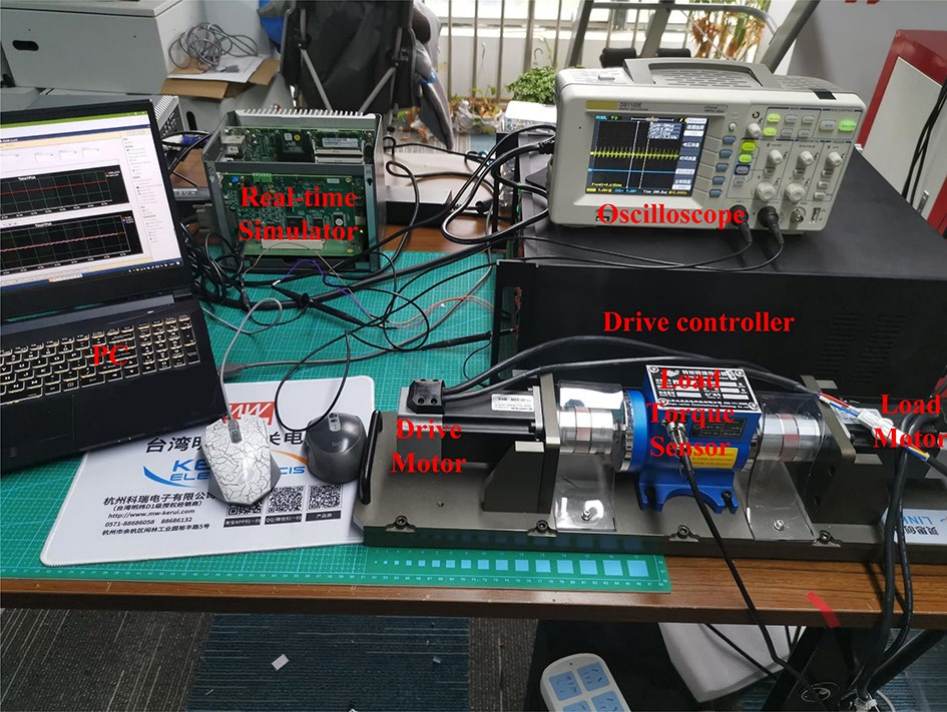
Experimental platform of the single-motor system.

**Figure 11:**
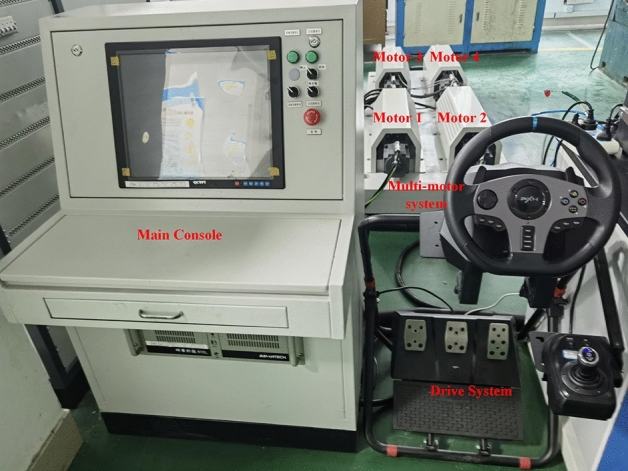
Experimental platform of the multi-motor system.

In this subsection, we build a single-motor control system to demonstrate the practical application of the proposed FTSMO NSMC, which is presented in Fig. 10. In addition, we build a multi-motor control system to observe the tracking performance between four motors, which is used to imitate the driving situation of four wheels in a DDEV, as shown in Fig. 11.

Because the PMIWM’s control performance is the same as the PMSM, we adopt the PMSM (made in China) with the same parameters to replace the PMIWM in order to verify the control performance. The motor system includes a control motor, a load motor, and a load torque sensor (Kilster, made in German). The control and communication parts consist of a host computer, a Links-RT simulator (POB1KAO, made in China), a motor-side driver, and a load-side driver. Signal instructions such as motor speed and torque are transferred through the host PC, and the real-time simulator sends control signals to the motor-side driver and load-side driver.

**Figure 12:**
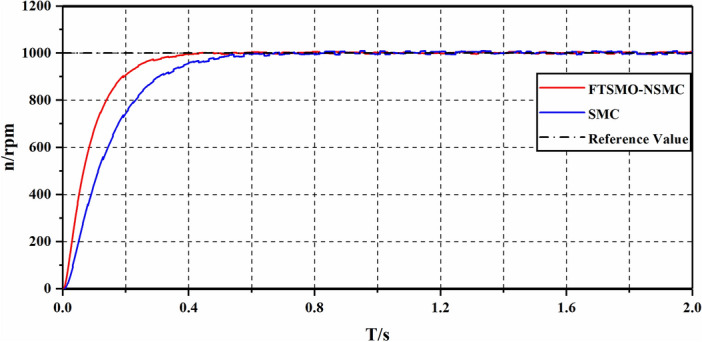
Experimental results of starting responses under the control strategies.

**Figure 13:**
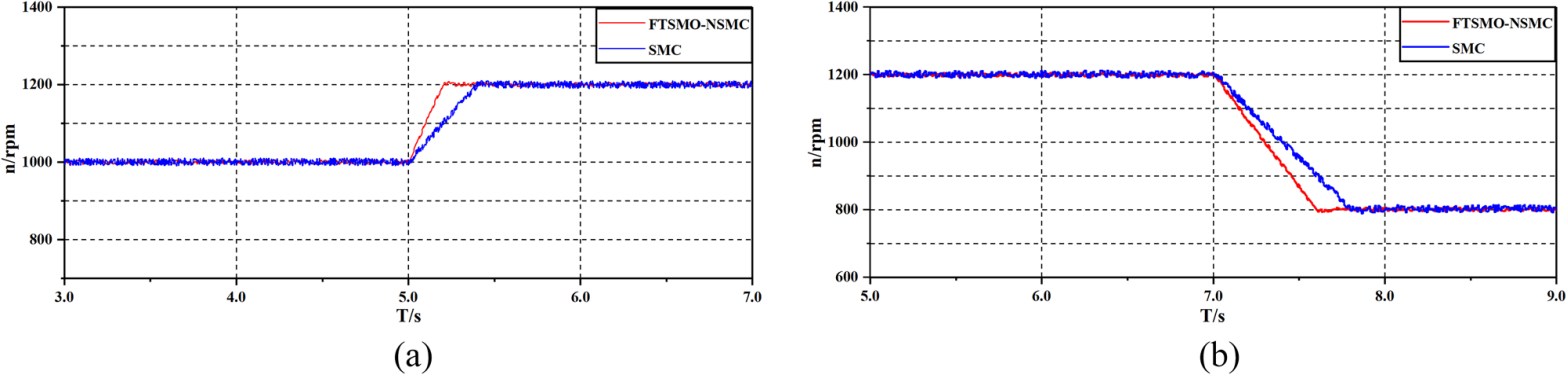
Experimental results of Speeding up and Slowing down (**a**) Speeding up(800 to 1200 rpm)(**b**) Slowing down(1200 to 800 rpm).

**Figure 14:**
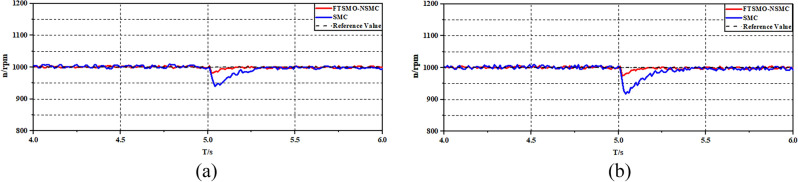
Experimental results of Speed responses under the control strategies (**a**) 10Nm (**b**) 20Nm.

**Figure 15:**
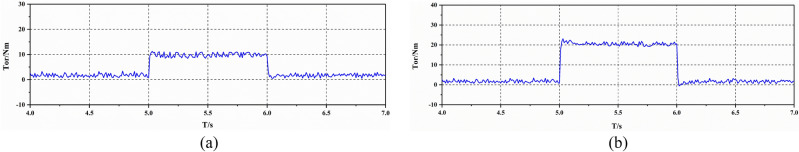
Experimental results of load torque observer when receiving torque command (**a**)10Nm (**b**) 20Nm.

In the first experiment, we implement experiments on the single-motor system to present the speed response performance under the FTSMO-NSMC and conventional SMC strategies. Figure 12 shows the starting performance of the strategies under the 1000 rpm instruction. Figure 13a,b illustrates the acceleration and deceleration response of the single-motor system under the FTSMO-NSMC and the conventional SMC strategies. Figure 14a,b shows the response performance when receiving different loading commands. Figure 15a,b presents the observed value.

As shown in Fig. 12, the proposed FTSMO-NSMC strategy can converge to the command speed at around 0.41 s, which is about 74% the value of the conventional SMC strategy. In addition, when arriving at the command speed, the speed variation of the proposed FTSMO-NSMC scheme is under 8 rpm, while the fluctuating value of the conventional SMC scheme is more than 4 times that of the proposed strategy. Therefore, the proposed FTSMO-NSMC has a better starting response and stability.

Figure 13a,b compares the response performance of the single-motor system when obtaining a 1000-to-1200 rpm acceleration instruction at 5.0 s and a 1200-to-800 rpm deceleration instruction at 7.0 s under the strategies of the FTSMO-NSMC and conventional SMC. As shown in Fig. 13, we can observe that the proposed FTSMO-NSMC consumes less time than the conventional SMC strategy when receiving the speed change command. In addition, the speed fluctuation of the conventional SMC is almost 3 times the value of the FTSMC-NSMC when the system reaches a steady state.

Figure 14a,b presents the anti-interference ability of the single-motor system when receiving a 10Nm and a 20Nm loading instruction, respectively. As shown in Fig. 14(a), when the motor receives a 10Nm external torque load instruction with a speed of 1000 rpm, the proposed FTSMO-NSMC scheme can save 43% time of the adjustment time with the comparison to the SMC scheme. In addition, the adjustment variation of the conventional SMC is almost 4 times that of the proposed scheme. Figure 14b can also verify that the proposed FTSMO-NSMC scheme has ideal robustness and dynamic performance.

Figure 15a,b shows different torque values observed by the FTSMO-NSMC scheme. When the control system receives a load instruction at 5.0 s, the proposed FTSMO can observe the load torque precisely and quickly, which shows that the FTSMO can effectively compensate for external load torque.

In the second experiment, the multi-motor system is adopted as the control object to validate the effectiveness of the disturbance compensation ability of the proposed FTSMO-NSMC scheme. As shown in Fig. 11, this multi-motor system consists of four motors installed in the DDEV. In addition, the moment of inertia of each motor can be adjusted by the inertia disk, and we set different moments of inertia for each motor to validate the parameter disturbance compensation ability of the proposed FTSMO-NSMC scheme. The moment of inertia of each motor in the multi-motor platform is set to J, 2 J, 5 J, and 10 J, respectively, corresponding to motors 1–4 in the multi-motor platform. In the following experiments, we give variable speed, loading, and unloading instructions to the multi-motor system and observe the tracking performance between motors, shown in Table 6.

**Table 6:** Three cases of different instructions.

Case	Instruction	Motor 1	Motor 2	Motor 3	Motor 4
1	0.0367	*J*	2* J*	5* J*	10* J*
2	0.52%	*J*	2* J*	5* J*	10* J*
3	0.003	*J*	2* J*	5* J*	10* J*

**Figure 16:**
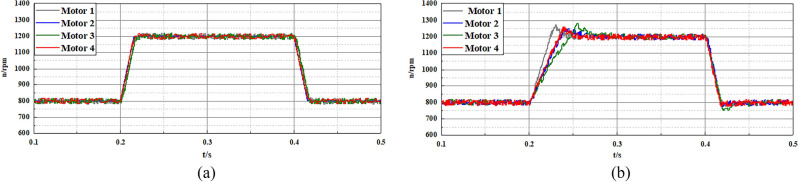
Experimental results of variable speed performance of multi-motor system (800–1200-800 rpm) (**a**) FTSMO-NSMC (**b**) SMC.

Figure 16a,b shows the speed tracking test of each motor receiving a speed change command of 800–1200-800 rpm when different moments of inertia J are set in the control platform of a multi-motor speed control system, which imitates acceleration and deceleration conditions of the four wheels of a DDEV. By comparing the two control strategies, it can be found that the proposed FTSMO-NSMC technology can achieve good speed tracking between each motor during motor acceleration/deceleration, and the response speed is faster than the conventional SMC. In addition, there is no overshoot phenomenon in each motor during the control process under the proposed FTSMO-NSMC scheme.

**Figure 17:**
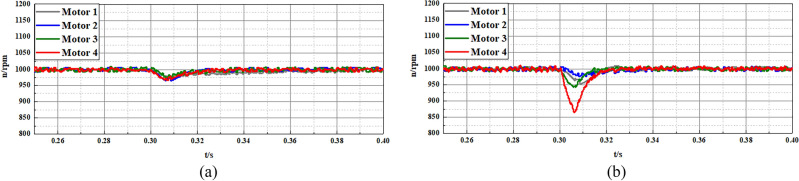
Experimental results of loading tracking performance of multi-motor system (10Nm) (**a**) FTSMO-NSMC. (**b**) SMC.

**Figure 18:**
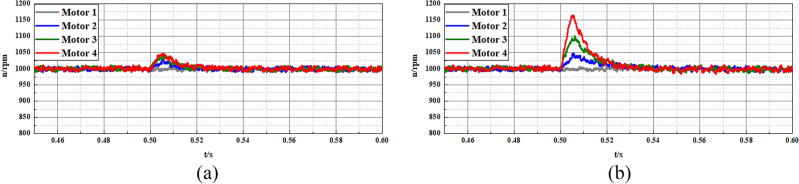
Experimental results of unloading tracking performance of multi-motor system (− 10Nm) (**a**) FTSMO-NSMC. (**b**) SMC.

Figure 17 and Fig. 18 present the anti-interference ability of the multi-motor system with different moments of inertia *J* when receiving 10Nm loading and -10Nm unloading instructions. As shown in Fig. 17 and Fig. 18, the proposed FTSMO-NSMC scheme presents a slighter speed tracking error than the traditional SMC. In addition, the proposed scheme has a better response speed and slighter adjustment fluctuation, which can improve the DDEV’s driving safety and stability when receiving disturbance interference.

## Conclusion

In this paper, we propose an FTSMO-NSMC strategy combined with a fuzzy control scheme to improve the control precision and reduce the negative impact of the parameter and load torque variation. The simulation and experimental results can draw the following conclusion:

1. Through the FTSMO proposed in this paper, the variation of parameter disturbance of the multi-motor system installed in the DDEV can be effectively compensated.
2. By designing the fuzzy controller and fuzzy rules, the control parameters of the NSMC scheme can be adjusted in real-time, which can further optimize the control precise and anti-interference ability of the motor system

In conclusion, the FTSMO-NSMC scheme can quicken the starting and response speed of the PMIWM system, as well as compensate for the influence of the parameter disturbance. Besides, the fuzzy controller can substantially eliminate speed fluctuation. Simulation and experimental results validate that the proposed method has the brilliant potential to be further applied to the DDEV industry. In addition, we install speed and torque sensors in the experiment platform, which are unsuitable for the DDEV that can undergo violent vibration and harsh environments. In future research, a sensorless control scheme will be designed to optimize the control stability of DDEV.

## Data Availability

The datasets generated and/or analysed during the current study are not publicly available due to the confidentiality requirements of our laboratory but are available from the corresponding author on reasonable request.
